# Proteomic Analysis of Human Sputum for the Diagnosis of Lung Disorders: Where Are We Today?

**DOI:** 10.3390/ijms23105692

**Published:** 2022-05-19

**Authors:** Maura D’Amato, Paolo Iadarola, Simona Viglio

**Affiliations:** 1 Department of Molecular Medicine, University of Pavia, 27100 Pavia, Italy; maura.damato90@gmail.com (M.D.); simona.viglio@unipv.it (S.V.); 2 Department of Biology and Biotechnologies “L. Spallanzani”, University of Pavia, 27100 Pavia, Italy

**Keywords:** sputum, proteomics, biomarker, COVID-19, COPD, cystic fibrosis, asthma, lung cancer

## Abstract

The identification of markers of inflammatory activity at the early stages of pulmonary diseases which share common characteristics that prevent their clear differentiation is of great significance to avoid misdiagnosis, and to understand the intrinsic molecular mechanism of the disorder. The combination of electrophoretic/chromatographic methods with mass spectrometry is currently a promising approach for the identification of candidate biomarkers of a disease. Since the fluid phase of sputum is a rich source of proteins which could provide an early diagnosis of specific lung disorders, it is frequently used in these studies. This report focuses on the state-of-the-art of the application, over the last ten years (2011–2021), of sputum proteomics in the investigation of severe lung disorders such as COPD; asthma; cystic fibrosis; lung cancer and those caused by COVID-19 infection. Analysis of the complete set of proteins found in sputum of patients affected by these disorders has allowed the identification of proteins whose levels change in response to the organism’s condition. Understanding proteome dynamism may help in associating these proteins with alterations in the physiology or progression of diseases investigated.

## 1. Introduction

Among the biological matrices that are informative of lung status, sputum has gained growing interest over the past decade for the investigation of several pulmonary disorders [1].

The rationale that makes sputum a wealth of information on lung status is that it contains a variety of components such as (i) mucus, (ii) microbial products from any colonizing bacteria/viruses, (iii) particulate/inhaled matter derived from the external environment, (iv) inflammatory cell components from cells resident within either the airways’ lumen or the tissue and, (v) cellular debris from all these compartments [2]. Although the first analyses of sputum date back to the early 1980s [3], only the technological advances in its collection and processing have made it possible to expand its applications. Highly reproducible measurements of cell counts as well as identification of type and severity of airway inflammation are currently performed on sputum of patients with chronic obstructive pulmonary disease (COPD) and bronchiectasis [4]. Likewise, accurate and sensitive analyses carried out on specimens from individuals with tuberculosis, cystic fibrosis, lung cancer, asthma, and chronic cough, [3,4,5] have improved our understanding of these disorders. Currently, one of the most challenging aims of sputum research is the identification of proteins whose level changes in response to the organism’s conditions. This may be achieved by means of “proteomics” i.e., the analysis of the complete set of proteins produced in a particular cell or tissue. Understanding proteome dynamism, in fact, may help to associate these proteins with alterations in physiology or progression of disease. Obviously, any tool that can produce accurate information about the pathological mechanisms of these disorders would offer a novel context for decoding the biological processes involved. Given the complexity of these systems, it is expected that only sophisticated technological techniques may provide suitable information on these potential markers. Two-dimensional electrophoresis (2-DE) and high-performance liquid chromatography (HPLC), both coupled with mass spectrometry (MS), have been platforms that perfectly responded to this need. In fact, their application to proteomics has resulted in substantial achievements in the screening of proteins as biomarkers of different diseases. Applied to different organs/tissues/physiological fluids, these strategies have made possible detection and identification of proteins that may be attractive for monitoring alterations in these districts [6,7,8,9,10,11,12].

The aim of this report is to provide an overview of the state-of-the-art of sputum proteomics for the study of selected lung disorders which, despite being a limited number, are the most representative of severe pulmonary infections. They include COPD; asthma; cystic fibrosis; lung cancer and lung pathologies caused by COVID-19. Advancements made over the last ten years in the identification of candidate biomarkers with a potential clinical utility are discussed.

## 2. Methodology Followed for the Preparation of the Current Article

To find the most relevant articles dealing with sputum proteomics related to the disorders considered in this manuscript, the criteria indicated below were followed. First, only articles in English were considered. Second, the research was focused on articles published in international journals in the last ten years (from 2011 to 2021) only. One article, although less recent, was selected because it was considered the “founder” of sputum proteomics (see ref. [7]). Third, systematic reviews, as well as classical articles were considered. The MeSH terms selected to perform a survey of the literature were “sputum biomarkers”, “proteomics”, “LC-MS/MS” coupled, from time to time, with the name of the lung disorder to be investigated i.e., SARS-CoV2; COPD; Asthma; Cystic Fibrosis and Lung Cancer. The full text of papers selected was analyzed, and repeated articles or those with redundant content were removed from the list.

**Figure 1:**
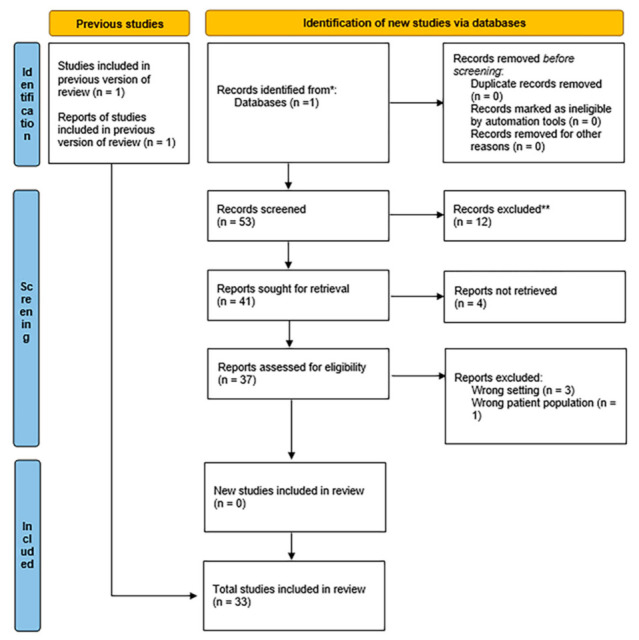
PRISMA flowchart that illustrates the procedure followed for the preparation of this review article.

Finally, two articles were selected for SARS-CoV2, fifteen for COPD, five for Asthma; seven for Cystic Fibrosis and four for Lung Cancer. The screening process is summarized in Figure 1, which illustrates the PRISMA flowchart. The aim of the PRISMA flow diagram is to record the number of articles initially found and then report on decisions made at various stages of the systematic review, thus making the selection process transparent. Numbers of articles are recorded at the different stages.

To make reading fluent, the current article is structured into paragraphs, each describing the application of sputum proteomics in investigating a specific lung disorder. The aim was to highlight the significant impact of this approach in biomarker development and future therapeutic in that specific field. Additional paragraphs outline the different technologies applied by the teams involved in this research as well as their challenges and/or limitations. The current information is placed in a future-facing context.

## 3. Sputum Collection and Manipulation

Sputum can be collected by a variety of procedures. Suctioning, consisting in the collection of sputum through an oropharyngeal or endotracheal catheter, is an invasive procedure not commonly used. It is usually applied to young pediatric patients or to patients who are intubated and cannot spontaneously produce a sample for laboratory analysis. In most cases, totally non-invasive (spontaneous sputum) or relatively non-invasive procedures are followed. Spontaneous sputum is inexpensive and easy to do. Patients are instructed to blow their noses and rinse their mouths out with water before expectorating sputum into a sterile container. In general, the presence of a health-care worker is desirable who supervises the patient during this procedure to avoid collection of saliva if the patient is not able to cough up sputum.

**Figure 2:**
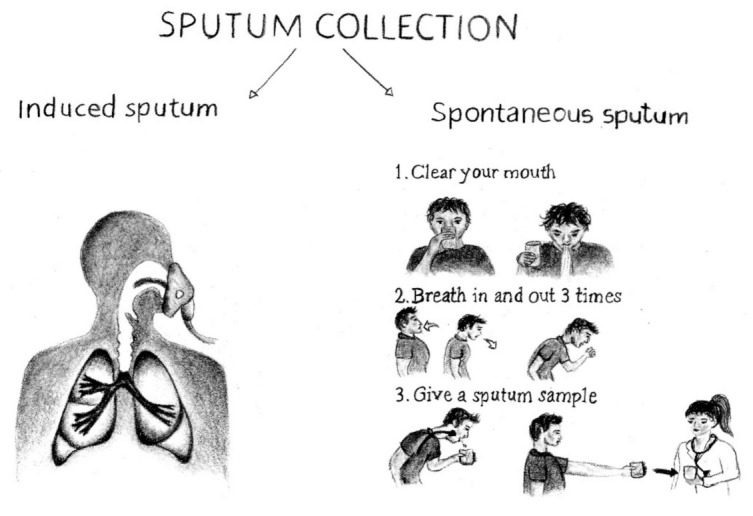
Cartoon showing the different procedures for induced (**left**) and spontaneous (**right**) sputum collection.

Induced sputum is collected by inhalation of nebulized hypertonic saline solution that liquefies airway secretions and promotes coughing [13]. This approach allows any individual to expectorate small amounts of sample and, to decrease even the minimal risk associated with induction, bronchodilators may be added to the saline solution [14]. The minimal discomfort is particularly appreciated by patients who must undergo repeated sampling in longitudinal follow-up studies [3,4]. Figure 2 schematically represents the difference between these two procedures. 

As for any other biological fluid, sputum requires some degree of sample preparation to assess the quality of specimens obtained. Despite being repeatable and inexpensive, sputum collection and handling may encounter several problems if not properly executed [13]. First, the number of steps in sample processing that are required by its peculiar feature (i.e., viscosity) may enhance the variability of sampling. Likewise, changes in sample dilution are critical and need data to be normalized. Moreover, great attention should be paid to the presence of contaminants (i.e., plasma, cells, microbes) that may interfere with the interpretation of data. For example, given that sputum must transit through the oral cavity, it can be contaminated by salivary proteins, which may also be common to sputum. This can be a problem as, to be representative of the material of interest, expectorant must exclusively come from the lungs; some salivary proteins are thus potential confounders [13]. Another major concern is the presence of abundant mucins, which are proteins characterized by high-molecular-weight/high charge that, through the formation of cross-links via sulfhydryl groups appear as large agglomerates that prevent conventional techniques from separating other sputum proteins [13].

## 4. Sputum Proteomics to Investigate SARS-CoV2

The enormous social impact this disease has caused over the past two years has led to the development of a great deal of research, proteomics playing a pivotal role in this field [15,16,17]. Obviously, most of this research focused on the detection of SARS-CoV-2 viral proteins in the nasopharyngeal and throat swabs that are used to collect virus specimens from COVID-19 patients [18,19,20]. The reason is that their collection is easy, and samples can be directly analyzed or grown in cell culture [18]. Nevertheless, since this disorder is characterized by a severe acute respiratory syndrome, it is also often accompanied by persistent cough. In this context, the results provided by patients’ sputum, complementary to those given by nasopharyngeal and/or throat swabs, allow to better explore the biological factors contributing to COVID-19 severity and to identify potential predictive biomarkers. In a very interesting methodological work, Bezstarosti et al. [21] demonstrated that targeted proteomics has the potential to identify SARS-CoV-2 proteins with sufficient sensitivity for clinical diagnostic labs in which mass spectrometry equipment is available. After assessing, by parallel reaction monitoring (PRM) MS, the limit of detection for several SARS-CoV-2 proteins in infected Vero E6 cells, they estimated that this limit for tryptic peptides of nucleocapsid proteins was in the mid-attomole range. The method was thus applied to the detection of viral proteins in COVID-19 patients using sputum and nasopharyngeal swabs as source materials for specimens. The PRM methodology allowed the authors to detect and relatively quantify SARS-CoV-2 tryptic peptides in all specimens analyzed. Moreover, the peak intensities in the mass spectra correlated well with the threshold cycle values obtained from PCR assays of the same samples. Based on these results the authors concluded that the technology based on PRM proteomics is suitable for the successful detection of viral infection in clinical specimens and can be used in clinical and diagnostics labs. An exciting article by Fisher et al. [22] demonstrates that sputum from severe COVID-19 patients contains neutrophil extracellular traps (NETs) consisting of extracellular DNA bound to neutrophil granule proteins that are released in response to bacteria as well as some viruses. In this study, the authors examined the sputum and blood plasma proteome from patients with COVID-19, applying a data-independent acquisition MS approach. This allowed identification of sputum NET-derived proteins which, by causing sputum thickening, cause significant hindrance to gaseous exchange during infections. Among these proteins, citrullinated proteins and acute-phase proteins associated with pulmonary inflammation were detected. The presence of NETs in the sputum was confirmed by immunofluorescence analysis and by ex vivo experiments in which DNase I was used to demonstrate that NETs could be degraded. This was the first study aimed at the characterization of the sputum proteome in patients with COVID-19. The novel finding that sputum of patients with severe COVID-19 contains NETs that can cause impairment of respiratory function provides important information on COVID pathophysiology and its possible recovery.

The summary of articles examined in this paragraph together with the methods used, the target of the research and the findings obtained are shown in Table 1.

## 5. Proteomics of Sputum in Chronic Obstructive Pulmonary Disease (COPD)

### 5.1. Stable COPD

Smoking is recognized as the most important causative factor of chronic obstructive pulmonary disease (COPD) and, in the absence of a specific cure, the most effective available treatment for COPD is smoking cessation. In fact, there is significant evidence that with smoking cessation the risk of developing COPD falls by about half. Although the complexity of this multi-factorial disease has provided a hurdle to the progress of research in this area, advances in proteomics observed over the past few years have shed new light on the mechanisms involved in smoke-COPD interaction. The forerunners of this research were Casado et al. [7] who, about 15 years ago, published an article dealing with the proteome profiles of sputum samples from smokers with lung disease whose severity ranged from normal (healthy smokers) to chronic obstructive pulmonary disease (COPD) with emphysema going through chronic bronchitis and COPD. Their data provided evidence that, from among the most highly expressed proteins identified in induced sputum (e.g., deleted-in-malignant brain tumor 1, DMBT1, secretory leukocyte protease inhibitor, SPLI, long palate lung nasal clone 1, LPLUNC 1, Mucin 5B, epithelial mucin 5AC), different proteins were expressed as the disease progressed from health to more advanced stages. This was the first relevant proof that distinct differences in protein expression profiles were related to the phenotype and cigarette smoking illness severity.

A deeper investigation into these proteins carried out a few years later by the same team of researchers [23] revealed that non-smokers could be discriminated from smokers based on the level of epithelial proteins and that Mucin 5AC (a protein of respiratory tract epithelia that protects the mucosa from infection and chemical damage by binding to inhaled microorganisms) was elevated in healthy smokers and chronic bronchitis. This suggested a continuum with the severity of hypersecretion determined by mechanisms of goblet-cell hyperplasia. In addition, sputum of emphysema subjects was unique in that it showed a high content of plasma proteins, histones and defensins, that are components of NETs. In contrast, defensins were correlated with epithelial proteins in all other groups. Finally, of great interest was the finding that network interactions were unique to each group [23]. Mucins have also been the target of the work by Reidel et al. [24], who investigated the potential adverse health effects of e-cigarettes on human airways by performing quantitative proteomics (with a nano-HPLC system) on induced sputum from smokers, non-smokers, and e-cigarette users. Both the total and the individual concentrations of MUC5AC and MUC5B were determined, the former by light scattering/refractometry and the latter by labelled mass spectrometry. The formation rates of NETs were also determined on the same subjects. The experimental results allowed the observation that the use of e-cigarettes altered the profile of innate defense proteins in airway secretions by inducing changes similar to those of cigarette smoking. In fact, elevated concentration of MUC5AC was observed in both cigarette and e-cigarette users, and the levels of innate defense proteins associated with COPD (i.e., human neutrophil elastase and matrix metalloproteinase 9) were significantly elevated in e-cigarette users as well. The increase of neutrophil granulocyte-related and NET-related proteins (i.e., myeloperoxidase, azurocidin, and protein-arginine deiminase 4) in sputum of e-cigarette users was also significant.

The relationship between cigarette smoking and the onset of COPD was also investigated by Titz et al [25] in a proteomic study performed by means of a nano LC-MS/MS system on sputum from individuals belonging to four different groups including never smokers, former smokers, current asymptomatic smokers, and smokers with early-stage COPD. As expected, the proteome of current smokers reflected the common physiological responses to smoke exposure, i.e., alterations in mucin/trefoil proteins and a prominent xenobiotic/oxidative stress response. By contrast, the group of former smokers showed nearly complete attenuation of these biological effects. Among the proteins identified, thirteen were differentially abundant between the COPD and the asymptomatic smoker group. These included TIMP1, APOA1, C6orf58, and LPLUNC1. Not surprisingly these findings were in good agreement with the results discussed above [7] and demonstrated that sputum profiling can capture the complex and reversible physiological response to cigarette smoke exposure. The same topic was investigated by Gao et al. [26] who were particularly interested in the association of the levels of bactericidal/permeability-increasing fold-containing protein B1 (BPIFB1) in sputum with smoking and with the longitudinal changes of lung function in smokers with COPD. Two-dimensional gel electrophoresis (2D-GE) and MS were used to characterize sputum BPIFB1, and its expression in COPD was investigated by immunoblotting and immunohistochemistry. The results of this research allowed the observation that secreted BPIFB1 was significantly elevated in the sputum of patients with COPD compared with that of smokers and non-smokers. In addition, the enzyme levels correlated with pack-years and changes in lung function over 4 years in current smokers with COPD. Based on these data, the authors concluded that BPIFB1 was one of the entities involved in the pathogenesis of smoking-related lung diseases.

Non-smokers, and smokers without and with moderate COPD were the subjects considered by Ohlmeier et al. [27] to identify proteins involved in COPD pathogenesis. Induced sputum from these individuals was analyzed by two-dimensional difference gel electrophoresis (2D-DIGE) coupled with MS. Among the proteins identified, 15 were differentially expressed, most of them being elevated in smokers and subjects with COPD. Polymeric immunoglobulin receptor (PIGR), a protein involved in specific immune defense and inflammation attracted the attention of the authors and a combination of complementary techniques (that included Western blot, immunohistochemistry/image analysis, and/or ELISA) was used to further investigate it, not only in sputum but also in lung tissue, and plasma. PIGR was characterized in sputum as a glycosylated secretory component. Upon examination of lung tissue, the level of this protein was significantly higher in the bronchial and alveolar epithelium of smokers compared to non-smokers. A further increase was observed in the alveolar area of mild to moderate COPD patients. Plasma PIGR was elevated in both smokers and smokers with COPD compared to non-smokers, and this increase was correlated with lung obstruction. To clarify the specific mechanisms of COPD, the same authors expanded their research to a cohort of patients with severe and very severe COPD (stage I-II and III-IV, respectively) and patients with α-1-antitrypsin deficiency (AATD) and idiopathic pulmonary fibrosis (IPF) [28]. The 2D-DIGE proteomic profiles obtained from lung tissue of these subjects were compared with those of non-smokers and smokers. The COPD-specific differential proteins were validated by immunoblotting and ELISA in induced sputum and plasma samples from the same patients. Among the altered proteins, COPD-, AATD-, and IPF-specific proteins were identified. Some others were overlapping or unspecific proteins. Cathepsin D, dihydropyrimidinase-related protein 2, tripeptidyl-peptidase 1, and transglutaminase 2 were validated as COPD-specific. While not being associated with smoking, this latter protein was found to correlate with COPD severity in lung tissue. In addition, the levels of transglutaminase 2 in sputum and plasma were elevated in patients with severe COPD and correlated with lung function. Due to these results, the applied procedure was considered suitable for the identification of new proteins related to COPD onset and severity. According to the authors, special attention should be paid to transglutaminase 2 as a potential diagnostic and therapeutic target for COPD.

The most exciting proteomic research advancing our understanding of COPD has been well discussed in two recent review articles [29,30]. In their report Moon et al. [29] explored proteins that have been consistently reported in the literature as the most promising blood and sputum biomarkers of COPD. The authors are confident that, with advancements in current mass spectrometry techniques and improvements in sequence database search, identification of new biomarkers can be enhanced. This will be helpful for filling in current gaps between biomarker discovery and patient care. The research progresses of protein biomarkers (in saliva and sputum) towards the management of COPD are also discussed by Dong et al. [30]. In particular, they investigated the promising clinical value of interleukins (IL-6 and IL-8), of matrix metalloproteinases (MMP-8 and MMP-9), of C-reactive protein, of tumor necrosis factor-alpha, and of neutrophil elastase. The possibility that clinically relevant biomarkers in saliva and sputum could be used for the future development of point-of-care sensors for chronic lung disease management was also explored by the authors.

### 5.2. Exacerbation of COPD

COPD patients may experience acute exacerbations of the disorder which consist of a worsening of respiratory symptoms due to acute inflammation and bacterial and viral infections [31]. This condition is recognized as a multi-systemic disease and is associated with an increase in the rates of morbidity and mortality. Given the poor knowledge of the pathways and molecules that drive acute exacerbation, identification of pathogenic molecules involved in the disorder will be useful in developing markers for this condition. To elucidate the role of different proteins in the development of acute exacerbations of COPD, numerous research groups performed their investigations on sputum (and other fluids) [32,33]. Assuming that the interleukin-1β (IL-1β) signaling pathway is implicated in COPD, the aim of the work by Damera et al. [32] was to explore the percentage of patients in which COPD is principally driven by activation of this pathway. To differentiate an IL-1β-associated sputum signature from other inflammation-associated COPD phenotypes, they performed a longitudinal study in which sputum samples from patients with stable-COPD and acute exacerbation were compared. A twofold increase of five IL-1β-mediated proteins (TNF-α, GCSF, IL-6, CD-40L, and MIP-1β) was observed in sputum of acute exacerbation patients relative to stable COPD state. The sputum levels of IL-1β did not show any significant association with known markers of other major COPD inflammation phenotypes. These findings confirmed the association between IL-1β pathway activation and airway bacterial infection in COPD.

To identify the substrates of proteases and to determine their activity in airways of COPD patients aimed at revealing disease mechanisms and potential treatments, Mallia-Milanes et al. [33] applied terminal amine isotopic labelling methodology (on a nano-HPLC system coupled to an LTQ-Orbitrap hybrid MS) on sputum samples from stable COPD patients and during exacerbations. Western blotting was used to assess fragments produced by specific protein degradation in airway samples. From among the numerous proteins identified in COPD sputum, several of them (including proteases and protease inhibitors, mucins, defensins, and other innate immune proteins) were proteolytically processed. Because of the production of increased amounts of airway neutrophils and neutrophil proteases during exacerbations, more proteins were cleaved at multiple sites. This was consistent with their degradation and inactivation. Moreover, during exacerbations different substrates, including protease inhibitors, mucins, and complement proteins were processed. These results allowed the observation that both the activity of airway elastase and processing of specific elastase substrates during stable disease differ from that in the phase of exacerbation.

### 5.3. Overlap of COPD and Asthma and their Differentiation

Although COPD and asthma are characterized by differences in lung function, acute exacerbations, and mortality, they share common pathological and functional features that make them overlap and even converge. The current differentiation between these multifactorial diseases is largely based on clinical and lung function parameters, due to their complexity, and they may often be misclassified. Paone et al. [10] were among the first to underline the important role of sputum and blood proteomics in the identification of biomarkers of airway inflammation that allow correct interpretation of disease and a more precise discrimination between patients. This concept was reaffirmed by O'Neil et al. [34] who, in a review article focused on the importance of identifying subgroups of disease entities to establish appropriate biomarkers and to enhance the understanding of underlying mechanisms in each subgroup, argued that advanced proteomics techniques have the inherent ability to provide a significant contribution to this cause. The work by Terracciano et al. [9,11,12,35] seems to fit this perspective. In fact, to elucidate the mechanisms underlying the pathobiology of COPD and asthma, they investigated the proteomic/peptidomic profiles of sputum from patients affected by these two disorders. First, they focused on the low-molecular weight proteome, the so-called peptidome [9]. These peptides were captured from sputum by means of a solid phase extraction procedure based on the use of both derivatized and non-derivatized hexagonal mesoporous silica beads. Their profile was obtained by applying a matrix assisted laser desorption ionization, time-of-flight (MALDI-TOF) MS platform. The comparison of COPD profiles with those of asthma patients and healthy controls allowed the authors to conclude that the chosen procedure could discriminate among them. In detail, six signals corresponding to three human α-defensins and three C-terminal amidated peptides (one of which was phosphorylated on serine), emerged as potential diagnostic peptidyl patterns able to differentiate these inflammatory airway diseases. Remaining within the scope of peptidomics, with the aim of improving the effectiveness of sample preparation protocols, these authors explored the benefits provided by another mesoporous silica sorbent [35]. Characterized by higher stability and thicker pore walls than the previous one [9], this material proved to be a versatile tool for peptidomic profiling of both sputum and synovial fluid, chosen as clinical sources of potential lung disease biomarkers. The peculiar features (pore size distribution, high pore volume and high surface) of sorbent beads allowed them to acquire highly enriched and well-resolved MALDI-TOF peptide profiles from the mentioned specimens.

These experiments demonstrated the suitability of hexagonal mesoporous sorbents in the selection of peptides with potential utility in a clinical setting from bodily fluids that can be difficult to analyze. The major issues related to sampling and processing procedures of proteomics-based investigations in the field of COPD/asthma performed on different specimens, including induced sputum, were highlighted by the same authors in two review articles [13,14]. The focus of these reviews was on the challenges of proteomic studies in discovering molecular targets potentially useful to improve and personalize the therapeutic management of COPD and to differentiate it from asthma. The proteomic profiles of induced sputum from healthy controls, COPD and asthma patients were also investigated by Zhang et al. [36] by means of ultra-performance liquid chromatography (UPLC) coupled with tandem MS. These profiles were compared with those obtained from airway mucus of severe COVID-19 patients. Their findings evidenced the presence of 455 differentially expressed proteins among the different groups investigated. Although differential proteins were found in COPD vs controls and asthma vs controls, of particular interest was the finding of 59 differentially expressed proteins specific to COVID-19 patients. From among these, 11 proteins were uniquely present in COVID-19 patients. As revealed by pathway and network enrichment analysis, eight of these (Complement component 9; Fibrinogen β and γ chains; Hemoglobin subunits α and β; Immunoglobulin ƛ; Proteinase 3) were overlapping proteins mostly associated with complement and coagulation cascades, platelet activation, or iron metabolism, and anemia-related pathways. As underlined by the authors, the usefulness of this study was to provide fundamental data for differentiating proteomic changes specific for COVID-19 patients from those related to COPD and asthma. This may suggest molecular targets for specialized therapy.

The profile of sputum proteins from thirty-one patients with various airway diseases, including COPD and asthma, was also explored by Dasgupta et al [37] using LC-MS/MS followed by sequential window acquisition of all theoretical fragments ion spectra (SWATH) for protein quantitation. This approach allowed quantification of a total of 185 proteins, 21 of which were identified and could distinguish between the clinical phenotypes by hierarchical clustering. Four groups were revealed: those that are inflammation related, oxidative stress-related, mucin-related and cytoskeletal and calcium-related. The levels of eight proteins (Azurocidin1, Neutrophil defensin 3, Lactotransferrin, Calmodulin 3, Coronin1A, Mucin 5B, Mucin 5AC and BPI fold containing family B1) were significantly altered (relative to mean) in exacerbator prone subjects compared to non-exacerbators. In addition, total protein concentration in sputum of frequent exacerbators was significantly higher than in other groups. It should be underlined that several of these proteins have already previously mentioned as potential biomarkers of COPD.

A schematic summary of all articles commented above is shown in Table 2.

## 6. Sputum Proteomics in Asthma

### 6.1. Asthma

As underlined above, asthma leads to increased irritability of the mucosa and the episodes of shortage of breath or coughing make this disorder overlap with COPD for a few traits [54]. The frequent observation of neutrophilic airway inflammation in both controlled asthma (CA) and severe uncontrolled asthma (UA) prevents the use of sputum biomarkers to differentiate the two conditions. Thus, to identify biomarkers of severe UA with neutrophilic airway inflammation, Lee et al. [38] applied two-dimensional electrophoresis coupled with MALDI-TOF/MS to the analysis of sputum samples from patients with CA and severe UA. From among the proteins that exhibited differences in relative intensity between patients of the two cohorts, the most impressive was S100 calcium binding protein A9 (S100A9), a calcium- and zinc-binding protein which plays a prominent role in the regulation of inflammatory processes and immune response. The fact that it was detected at a higher level in neutrophilic sputum from patients with severe UA vs CA led the authors to hypothesize that sputum S100A9 could be considered a potential biomarker of neutrophilic inflammation in severe UA.

Non-smokers, healthy non-smokers, ex-smokers (ESA) and current smokers (CSA), were the components of the cohorts of patients examined by Takahashi et al [39] to define severe asthma molecular phenotypes. The broad range of analyses performed by the authors included exploratory proteomic analysis of sputum supernatants and transcriptomic analysis of bronchial brushings, biopsies, and sputum cells. Based on the differentially expressed proteins identified, the usefulness of sputum proteomics in distinguishing CSA from ESA subjects was reaffirmed despite little difference in their clinical characteristics. The peculiarity of this difference was an increase of the expression of colony-stimulating factor 2 protein in CSA patients and a remarkable loss of epithelial barrier processes in ESA patients. Based on prespecified clinic-physiologic variables, Lefaudeux et al. [40] could compare cohorts of patients with moderate-to-severe asthma and stratify them into four clusters (Table 2 and Table 4, respectively) that showed significant differences in sputum proteomics and transcriptomics. The data relative to severe asthma clusters (Table 2, Table 3 and Table 4) indicated that sputum eosinophilia was higher than in cluster T1, while no differences was observed in sputum neutrophil counts and exhaled nitric oxide and serum IgE levels.

The improvement of patient stratification by the identification of molecular sub phenotypes of asthma defined by proteomic signature was undertaken by Schofield et al. [41]. They applied an unbiased label-free quantitative MS technique combined with topological data analysis to analyze the proteomes of sputum supernatants from asthmatic patients. Based on similarity in proteomic features, patients were stratified in ten clusters representing three sub-phenotypes of asthma: highly eosinophilic, highly neutrophilic, and highly atopic with relatively low granulocytic inflammation. The insight on granulocytic inflammation provided by these data could be useful for detection of targets for novel therapies.

### 6.2. Asthma and Gastro-Esophageal Reflux

Gastro-esophageal reflux disease (GORD) is one of the numerous co-morbidities of asthma. Despite being clear that there is a significant association between these two disorders, the paucity of data on the direction of causality makes the role of GORD poorly understood [55]. In this context, based on the assumption that severe asthmatics with active GORD inhale oropharyngeal refluxate into their lower airways where it causes severe biological problems, Tariq et al. [42] performed a proteomic analysis on induced sputum of mild/moderate asthmatics and healthy controls and of a subset of severe asthmatics. Quantitative LC coupled with untargeted MS was used to perform the experiments, and proteins associated with GORD in the cohort of severe asthmatics were identified by means of univariate and multiple logistic regression analyses. The data evidenced that GORD was three- and ten-fold more prevalent in severe asthmatics compared to mild/moderate asthmatics and healthy controls, respectively. A further comparison of the sputum proteome in active GORD severe asthmatics with that of patients without active GORD showed five differentially abundant proteins with roles in anti-microbial defenses, systemic inflammation, and epithelial integrity. Multiple linear regression analysis revealed that three of these (Ig lambda variable; plasma protease C1 inhibitor, and lipocalin-1) were associated with active GORD. The evidence provided by this study indicates that severe asthmatics with GORD may represent a distinct phenotype of asthma, and that reflux can cause subtle perturbation of proteins detectable in the airways lining fluid.

The list of articles commented in this paragraph is summarized in Table 3.

## 7. Sputum Proteomics to Investigate Cystic Fibrosis

Cystic fibrosis (CF) is a lung disease characterized by chronic inflammation caused by high levels of cytokines and chemokines in the airways. As underlined in previous paragraphs, the rationale for using sputum proteomics to study these disorders is that monitoring the expression of biomarkers predictive of pulmonary infection and/or exacerbation would potentially prolong the survival of CF subjects. In this context, the work of Sloane et al. [43] aimed at elucidating changes in protein profiles and expression as markers of disease progression. They used 2-DE coupled to MALDI-TOF MS to compare sputum protein profiles from exacerbated CF subjects with (i) a subgroup of the same subjects after hospital treatment, (ii) clinically stable CF children with preserved lung function, and (iii) control subjects. Interestingly, two children with CF showed profiles and biomarker expression resembling those of exacerbated adults during an exacerbation. In addition, the levels of differentially expressed myeloperoxidase, cleaved α1-antitrypsin, IgG degradation, interleukin-8, and total protein concentration, together with their correlation to Forced Expiratory Volume in one second (FEV1), were statistically significant. Changes in myeloperoxidase expression and IgG degradation were indicated by statistical correlation analyses as the strongest predictors of FEV1.

An alternative biomarker discovery strategy involving an immunocapture method in which circulating antibody repertoires from plasma of CF patients are immobilized and subsequently used to capture antigens from complex proteomes of sputum or bacterial extracts, was explored by the same authors [44]. Captured antigens were separated by 2-DE to obtain the resolution of protein isoforms and perform multiple analyses (including MS-based identification and characterization) of each captured protein. Furthermore, the use of a chemical inkjet printer allowed them to demonstrate how gel-extracted protein aliquots can provide instant validation of protein immunogenicity across multiple clinical samples. Thanks to the application of this new immunoproteomic strategy, the authors could isolate from sputum of CF subjects with acute exacerbations a variety of chronic inflammation-associated autoantigens (e.g., enolase 1B, myeloperoxidase, the regulatory subunit calgranulin B of the CF antigen complex and antigenic fragments of IgG heavy chains). Bacterial proteins from P. aeruginosa involved in the anaerobic stress response and alginate synthesis were also identified. These results allowed the authors to affirm that this immunocapture method could be an important tool for expanding the repertoire of biomarkers available for the development of point-of-care tests that can be used for rapid and accurate diagnosis of disease.

To identify potential biomarkers of suppurative and inflammatory lung disease, Gray et al. [45] analyzed by surface-enhanced laser desorption/ionization time-of-flight (SELDI-TOF) MS the fluid phase of induced sputum collected from healthy subjects and patients with (i) asthma, (ii) COPD, (iii) CF, and (iv) bronchiectasis. Sputum of a few CF patients was sampled before and after antibiotic therapy for an infective exacerbation. Potential biomarkers that differentiated each of the disease groups from healthy controls were identified as calgranulins A, B, and C, Clara cell secretory protein, lysozyme c, proline rich salivary peptide, cystatin s, and hemoglobin a.

To obtain insight into the pathophysiology of the microbial community in lungs of CF patients, Graf et al. [46] developed a reliable and widely applicable protocol for sputum processing, microbial enrichment, cell disruption, and protein extraction. A detailed metaproteome analysis was also performed on a restricted number of CF sputum samples. The results suggested that the contribution to the CF pathophysiology of the arginine deiminase pathway and of multiple proteases and peptidases identified from various bacterial genera could have been under appreciated by scientists. For this reason, the authors concluded that their study could represent an important basis for future investigations on the physiology of microbial pathogens in CF in vivo. This is an important prerequisite for the development of novel antimicrobial therapies to combat chronic recurrent airway infection in CF. Sputum cellular proteins in CF adults chronically infected with P. aeruginosa (PA) were identified by Pattison et al. [47] using a sophisticated platform consisting of multi-dimensional protein identification technology (MudPIT) together with Ingenuity Pathway Analysis and Gene Ontology to evince protein abundance, their potential clinical significance and their correlation with lung function. The CF proteome was largely distinct from that of healthy subjects, the proteins involved in immune functions (including neutrophil recruitment, rearrangement of cytoskeleton, phagocytosis and T-cell signaling) being among those predicted as most upregulated in CF. Matrix metalloproteinase-8, annexin I and nicotinamide phosphoribosyl transferase, implicated in delayed apoptosis and clearing of inflammatory and immune cells, were also differentially abundant CF-associated proteins. Very intriguing was the finding of six proteins, consistently abundant in CF patients when compared to healthy controls, known to be constituents of NETs. Several proteins consistently detected in CF sputum correlated negatively with lung function (measured as Forced Expiratory Volume in the first second). This finding suggested that a variety of parameters, including the neutrophil influx, protease activity, the degree of inflammation, and airway remodeling, may contribute to defining lung function.

*P. aeruginosa* physiology in patient airways was also investigated by Wu et al. [48] to better understand how in vivo bacterial functioning differs from in vitro conditions. The PA proteome was analyzed in vivo in sputum samples from CF patients following an experimental approach that consisted of a novel bacterial-enrichment method relying on differential centrifugation and detergent treatment. The LC-MS/MS data were then compared with those from ex vivo-grown *P. aeruginosa* populations belonging to the same patient. This approach revealed the in vivo up-regulation of siderophore TonB-dependent receptors that caused a remodeling in central carbon metabolism, including glyoxylate cycle and lactate utilization, and alginate overproduction. This information was considered of great interest as it could lead to the development of future treatment strategies aimed at altering PA physiology in vivo to compromise infectivity or improve antibiotic efficacy.

Several PA isolates from CF patients’ sputum were sequenced by Penesyan et al. [49] and compared to each other and with the model strain PAO1. A 1-DE and MS proteomic analysis revealed that, in a conventional medium, PAO1 expressed numerous proteins that were absent in the CF isolates. These latter shared a distinctive signature set of proteins not detected in PAO1. In fact, while PAO1 expressed many transporters for the uptake of organic nutrients and relatively few biosynthetic pathways, the CF isolates expressed a narrower range of transporters and a broader set of metabolic pathways for the biosynthesis of amino acids, carbohydrates, nucleotides, and polyamines. These proteomic data demonstrated that while in a common medium PAO1 may transport different nutrients from rich medium, the CF isolates may only utilize a limited number of nutrients from the medium, relying mainly on their own metabolism for the synthesis of essential nutrients.

An opportunistic pathogen that affects patients with diabetes, solid tumors, chronic lung diseases and stem cell transplants is *Scedosporium aurantiacum*, the second most common filamentous fungus (after Aspergillus fumigatus) isolated in Australia from the sputum of CF patients [56]. For the first time, Han et al. [57] explored the proteases secreted by *S. aurantiacum* to study their potential roles in fungal virulence. The amount, type, and activity of major proteases secreted by a clinical isolate and an environmental strain were determined in response to cultivation on synthetic CF sputum medium supplemented with mucin or casein. Six homologs of fungal proteases were identified from the clinical isolate and five from the environmental one. Three protease homologs, including a subtilisin protease S8, a putative leucine aminopeptidase and a PA-SaNapH-like protease, were common for both isolates. The list of articles discussed in this paragraph is summarized in Table 4.

## 8. Sputum Proteomics and Lung Cancer

Lung cancer is a life-threatening disease whose onset is related to numerous risk factors, among which cigarette smoking plays a very important role [57]. It is currently considered the leading cause of cancer mortality in the world, and, in most cases, its cure is difficult/impossible due to the appearance of symptoms when the disease is in an advanced state or metastatic [58]. It is thus easy to understand how crucial the early screening of the biological factors that lead to the development of the disorder is. As for other disorders, a strategy that can contribute to a better understanding of disease biology is sputum proteomics. In a very interesting work, Yu et al. [50] demonstrated the importance of targeted proteomics to the identification of proteins involved in lung cancer with sufficient sensitivity for clinical diagnostic laboratories in which mass spectrometry equipment is available. Sputum supernatants from lung cancer patients and healthy controls were analyzed in parallel with proteins separated by SDS-PAGE. Bands were then excised, digested with trypsin and peptides separated by LC-MS. The expression levels of five proteins (including ENO1, the membrane protein DAP10, the nucleotide exchange factor guanine, the tumor cleared protein related to low-density lipoprotein receptor and hemopexin) were higher in sputum of cancer patients compared to controls. Significantly, the level of ENO1 was approximately four times higher than that of other proteins. These results agreed with previous observations [57], thus confirming ENO1 as the first major early-stage lung cancer biomarker. A comprehensive discussion about the importance of relying on molecular biomarkers for the early detection of lung cancer can be found in the review article by Hassanein et al. [59]. To establish a new diagnostic and prognostic biomarker, Ali-Labib et al. [51] focused their attention on metalloproteases (MMPs). The significant roles of MMPs in the metabolism of extracellular matrix components, tissue repair and cell migration are well-known [60]. Their importance as biomarkers consists in the role they play in tumor invasion and metastasis and in the involvement in the early stages of tumor development by regulating cell proliferation, apoptosis, angiogenesis, and immunity [61,62]. In this work, the quantitative analysis of MMP-2 in sputum was performed by ELISA assay. The results showed a marked increase of their levels in the malignant group compared to the benign and control groups. Furthermore, MMP-2 increased exponentially with respect to the severity degree of the disease. Finally, a significant increase in the level of MMP-2 was observed in the sputum of metastatic compared to the non-metastatic group. Taken together, these data strongly support the hypothesis that this molecule could be a biomarker of the disease. Another important work, by Rostila et al [52], was aimed at recognizing potential biomarkers of lung cancer by validating a series of proteins known for their connection with oxidative stress and production of reactive oxygen species (ROS). The amount of ROS in cells is tightly regulated by the 2-related p45 erythroid nuclear transcription factor 2 (NRF2) whose uncontrolled expression leads to the production of high levels of peroxiredoxin (PRX) and thioredoxin (TXN) [63]. Although PRX and TXN exert a protective and stabilizing role (by reducing H2O2 to water and oxygen), the overexpression of PRX has been associated with tumor progression [64]. In this study, 2D-DIGE coupled with LC-MS/MS was used to detect protein abundance differences in the proteome of induced sputum obtained from smokers, subjects exposed to asbestos, and lung cancer patients. Five proteins, four of which are associated with lung cancer (PRDX2, TXN, GAPDH and S100A8), were validated. The abundance of the fifth protein (ECM1), while being implicated in many cancerogenic processes, was not significantly different among cohorts. The identification of patients who are most likely to respond to platinum-based lung cancer chemotherapies was addressed by the study of Böttger et al [53]. SDS-PAGE followed by nano LC-MS/MS yielded a list of 58 proteins, among which two (UDP-glucose glycoprotein glucosyltransferase 1 and collagen chain alpha-1 (VI)) were best biomarker candidates for sensitivity to cisplatin, and one (protein 4 associated with microtubules) was the best biomarker candidate for resistance to cisplatin.

A schematic summary of all articles discussed above is shown in Table 5.

## 9. Proteomics of Extracellular Vesicles in Lung Disorders

Although no specific articles dedicated to the proteomics of sputum-derived extracellular vesicles (EVs) were found in the literature, the important role played by these structures in intercellular communication and possible diagnostic markers of disease is worthy of mention.

By transferring molecules (including RNA, lipids and proteins) to recipient cells, EVs have the promise of being multimodal biomarker candidates of disease, particularly in response to respiratory stressors in COPD, CF, asthma, lung cancer and idiopathic pulmonary fibrosis. Obviously, due to their potential as drug carriers, EVs are on the horizon as new modes of drug delivery and as therapies themselves in cell-based therapeutics.

The potential of EVs as markers for disease severity has been demonstrated by their proteomic profiling that allowed the successful identification of markers of acute exacerbation in COPD and unique protein fingerprints at different ages in persons with CF [65,66]. Moreover, MS analysis of EVs successfully identified differential proteomes in patients with lung adenocarcinoma [67], and unique proteomes were exhibited by EVs isolated from nasal lavage fluid of asthmatic patients compared with controls [68].

An excellent review article dealing with the role of extracellular vesicles in chronic lung diseases has been recently published by Trappe et al. [69].

## 10. Exploring the Role of Biomarkers

Although limited to the analysis of sputum, the content of the above paragraphs shows that a significant fraction of proteomic investigations in the field of lung disorders is focused on the search for biomarkers that could either make their diagnosis more precise or identify the outcomes of the disease itself. There is a firm belief that biomarkers hold real promise for advancing the way we treat certain diseases (e.g., COPD) and the large body of literature in this area documents this interest [70,71,72]. Let us dwell for a moment on the concept of a biomarker. According to the definition of Group [73], the term biomarker refers to “the characteristics or signs that are measurable and as such could indicate the normal or pathogenic biological processes or responses to treatment”. This is the rationale for an urgent need to identify reliable biomarkers for the early diagnosis (as well as to monitor whether a particular treatment is beneficial to an individual) of the lung disorders considered in this report. The reasons for emphasizing this urgency are at least three. First, these are aggressive malignancies whose incidence has increased worldwide in recent decades and is expected to continually rise. Second, these pathologies generate substantial costs for the health system, mainly related to hospitalizations and the associated pharmacological treatment. Finally, not to be underestimated are the indirect costs of the disease (i.e., within the profile of COPD/asthma patients) due to the loss of productivity and premature retirement of patients. The importance of these aspects is highlighted by the vast amount of literature dealing with the magnitude of resources needed to treat patients with these severe disorders, and with the public health measures taken by the world governments to face their impact on human psychology, the educational system, and the global economy [74,75,76,77,78]. How can proteomics alleviate these issues?

The variety of articles discussed above points to this methodology as one of the possible routes for the identification of potential biomarkers that could prove beneficial in discovering biochemical signatures involved in disease etiology.

Regardless of the strategy applied, all studies have shown the presence of several down- or up-regulated proteins between healthy and diseased and/or among patients with different levels of disease severity. Interestingly, some alterations in the expression of specific proteins could be (positively or negatively) correlated to lung function, thus demonstrating that these changes (protein accumulation/decrease, mis-localization) could be responsible of protein toxicity with consequent downstream processes ranging from inflammation to cell death. If these proteins prove to be robust biomarkers, unravelling their structure and function (and, possibly, their interactions with other proteins) becomes crucial for moving forward to a deeper understanding of these diseases. However, though promising, proteomics has many challenges to overcome and is not able to explain disease development fully. Thus, assuming that these proteins (or some of them) are biomarkers of certain disorders why have only a few of them (if any) been validated for routine clinical practice? In fact, while proteomics, metabolomics and DNA microarrays are included in a voluminous literature documenting hundreds of claimed biomarkers, several deficiencies in this literature make it difficult to fully evaluate the performance of these biomarkers. Basically, so far very few have allowed to establish robust correlations with patient's health status or responses to treatment [79]. For example, blood fibrinogen was likely the first COPD biomarker presented to the Food and Drug Administration for qualification in the drug approval process [80,81]. Together with C-reactive protein, it has been associated with COPD and, in some instances, future risk of developing COPD in targeted populations [80,81]. More recently, the role of DNA methylation was explored as a potential biomarker for COPD prevention, diagnosis, and prognosis [82]. The great enthusiasm about these successes gives hope that the way is now open, although many questions about interpretation of results on COPD biomarkers remain unanswered [83]. Likewise, if exhaled nitric oxide and blood eosinophils have definite clinical value as biomarkers of allergic asthma in non-smokers [84], further studies are needed to confirm the clinical utility of relevant biomarkers currently used in asthma [85,86].

In conclusion, there is a gap (obviously not only confined to lung disorders) that has thus far delayed the translation of the most promising results in biomarker discovery from an academic laboratory to the clinic. This gap reflects the failure of researchers to adopt standardized procedures. For example, patients are not always matched for as many variables as possible (i.e., age, sex, weight, ethnicity, smoking and alcohol use, previous pharmacological treatments received). In addition, methodological heterogeneity, the lack of standardization among studies, panels of proteins not replicable, or only partially replicable with almost no overlap across studies, are all factors that often prevent the generalizability of the results. To increase the reliability and reproducibility of data, measures should be harmonized, and inter-study heterogeneity mitigated through a standardized operational procedure. Given the complementary strengths of different technologies (transcriptomics, metabolomics, and proteomics), only their combination may provide a complete picture of a complex metabolism. This could be a way to create more robust data that allow to correlate biomarkers with people's conditions and responses to treatments.

## 11. Methodological Considerations in Sputum Proteomics

Reviewing previous paragraphs, it can be observed that both in-gel and off-gel approaches have been coupled with mass spectrometry for protein identification. Obviously, the expression “in-gel technique” in proteomics refers (with few exceptions) to 2-DE, the most common gel-based approach utilized in this field due its robustness and reliability. While being fundamental to the birth of proteomics [87], 2-DE has been apparently superseded by the astonishing rate of liquid chromatographic and bioinformatic technologies advances observed over the last two decades. Despite the intrinsic problems (ranging from poor representation of low abundant and hydrophobic proteins to difficult detection of proteins with extreme molecular size or pI) that limit the resolving power of this technique, the numerous works discussed in this report (see refs. [26,43,44,49]) and a variety of recent review articles [87,88,89,90,91] seem to overturn the current common belief that 2-DE is an outdated method. Based on the results published by these authors, this technique still has some merit, being, in a few cases (e.g., quantify differences in protein expression between samples from healthy vs controls, see [38,43], or in the separation of protein isoforms, see ref. [44]), preferable to the off-gel approaches. A significant contribution to the overcoming of these limitations has come from the development of two-dimensional fluorescence DIGE (see refs. [27,28,52]) that allows reduction of gel-to-gel variations, thus improving the analytical power of gel-based methods. With its highly accurate quantitative dimension, DIGE represents the ultimate evolution of conventional 2-D electrophoresis, enabling multiple protein extracts to be separated on the same 2-D gel and allowing differentially expressed proteins to be easily detected. In the light of the works considered in the previous paragraphs, it can be stated that, although limited, the application of 2-DE to sputum proteomics has contributed (and, perhaps, it will continue to do so) to disclose both proteins and physiological mechanisms associated with clinical pathologies that can aid in the discovery of biomarkers of lung disorders. While acknowledging these merits of 2-DE, it should be recognized that most of the works described above were done using liquid chromatographic platforms ranging from HPLC (see ref. [39,42,48]) to UPLC (see ref. [36,37]), capillary/nano LC (see ref. [7,23,24,25,33]) and MudPIT (see ref. [47]). The intrinsic properties of these technologies allowed researchers to profile with considerably greater dynamic range of coverage the proteome of samples investigated, in all cases a high number of differentially expressed proteins, including low abundant ones, being confidently identified.

Obviously, independently of the chosen strategy (in-gel or off-gel), MS analysis represents the key step for the identification and characterization of proteins. In most sputum proteomics works here considered, soft ionization techniques capable of generating stable gas phase ions from thermally instable molecules, and to identify and/or quantify proteins isolated from biological fluids and tissues [92], MALDI and electrospray ionization (ESI) have been used. In one case (see ref. [45]) SELDI-TOF was applied to differentiate cohorts of patients affected by different lung disorders. Despite being widely criticized in the literature due to the poor reliability of data [93], in some cases this approach is still applied with the intention to generate a potential “panel” of biomarkers with higher diagnostic sensitivity and specificity compared to other techniques. This approach relies on interpretation of mass spectral patterns rather than identifying individual proteins and is used to generate ‘‘protein fingerprints’’.

Thus, although the evidence that in-gel and off-gel platforms have many points of comparison and contrast, it can be inferred that both strategies can resolve hundreds to thousands of proteins in sputum samples analyzed. The works discussed in this report suggest that the biological question addressed often directs the choice between the different platforms.

## 12. Potential New Modalities of Sputum Analysis with Clinical Relevance

Are there novel approaches for the analysis of sputum that may provide important information for the diagnosis and management of pulmonary disorders? Most of the articles discussed in previous paragraphs described comparative studies aimed at the capture of differentially expressed proteins in different cohorts. However, this is only one of the possible options of proteomics. While providing a significant contribution in the research of key players in various conditions, this step only gives direction for prioritizing proteins of interest to be investigated in further studies from various perspectives. In fact, the options offered by proteomics include the study of the interactions with other proteins, the subcellular localization of proteins, their tissue-specific expression, and their half-life. With the contribution of the most advanced statistical methodologies, all together these data will improve the understanding of mechanisms underlying these severe lung disorders.

In addition to LC-MS, the focus on the use of Nuclear Magnetic Resonance (NMR) to perform detailed sputum metabolomic analysis in lung disorders, with particular emphasis to CF, seems to be growing [94,95]. Given its advantages in terms of quantification of activation products of inflammatory cells and of measurements of total and differential cell counts (which is the usual approach in basic cellular analysis of sputum), NMR is one of the most powerful approaches for metabolomic analysis. Providing evidence of inflammatory cell activation, NMR analysis of sputum will be helpful to clarify the contribution of eosinophils to the inflammatory processes in CF [95]. The application of this analytical technique to a wide range of biological samples (tissues, biological fluids, cells) has made it the preferred platform for large-scale clinical metabolomic studies [96]. The possibility to extend this application to sputum of patients affected by lung disorders could allow detection and characterization of compounds not easily identifiable with LC-MS (e.g., organic acids, alcohols, sugars, and highly polar compounds in general).

Another aspect that could be of topical interest in future studies on sputum from these patients is the relevance of microbiota for pulmonary health and disease. The relationships between microbiota in samples taken directly from the lung and the microbiota in spontaneously expectorated sputum has been explored in a number of studies focused on CF and COPD [97,98,99,100,101]. The results seem to suggest that the composition of the microbial community in the lung may influence pulmonary health and vice versa. Recently it has also been demonstrated that the sputum microbiome of healthy subjects is distinct from that of COPD patients, and that is independent of smoking history [102]. While being in an early stage, there is an increasing body of evidence indicating that the respiratory system microbiome in normal healthy subjects presents a density and a high diversity of bacterial colonies from those observed in pathological conditions. As mentioned above, if promising biomarkers arise, effective strategies to test whether the use of microbiome data can affect clinical outcomes are needed.

## 13. Concluding Remarks

Currently ‘‘OMICs’’ platforms are the most informative techniques which may help scientists deciphering the molecular mechanism of lung infections characterized by a particular complexity. If integrated with each other, genomics, metabolomics, lipidomics, and proteomics may provide significant results in clinical research. What appears from the analysis of the numerous articles considered in this report is that proteomics of sputum is a valid tool to evidence protein alterations which could shed new light on the understanding of severe lung disorders. Collection of sputum is easy, not expensive, and non-invasive and it can be repeated without causing discomfort to the patient in case of longitudinal studies. Identification of proteins in this fluid proved to be helpful for the differentiation of healthy from patients and of patients with the same disorder at different levels of severity. For example, different proteins were expressed in COPD patients as the disease progressed from health to more advanced stages. Another study revealed that non-smokers could be discriminated from smokers based on the level of epithelial proteins. TIMP1, APOA1, C6orf58, and LPLUNC1 were proteins differentially abundant between COPD patients and the asymptomatic smokers. In addition, sputum of emphysema subjects was found to be unique in that it showed high content of plasma proteins and of histones and defensins, which are components of NETs. Based on the proteomic/peptidomic profiles of sputum from patients affected by COPD and asthma, these two disorders could be discriminated at a molecular basis. Moreover, the CF proteome was found to be largely distinct from that of healthy subjects, the proteins involved in immune functions (including neutrophil recruitment, rearrangement of cytoskeleton, phagocytosis and T-cell signaling) being among those predicted as most upregulated in CF. Fundamental data for differentiating proteomic changes specific for COVID-19 patients from those related to COPD and asthma, thus suggesting molecular targets for specialized therapy, have also been provided by sputum proteomics. In addition, protein panels that could be markers for the diagnosis and/or prognosis of lung cancer and prediction of responses to therapy have been characterized. However, the question of whether the molecules underlined in these reports can be considered markers of disease remains open, most of them not being validated. What is encouraging is that experimental work carried out by different research teams in distant laboratories have often produced the same results. Thus, focusing on these proteins could be the safest route to transfer quickly our knowledge from the lab to the bedside. It can be concluded that sputum proteomics is making a good contribution to opening horizons in clarifying various aspects of these infections.

## Figures and Tables

**Table 1:** List of articles on proteomics of sputum in COVID -19 patients.

Subjects Investigated	Method of Sputum Collection and Processing	Proteomic Technique Applied	Target of the Research	Finding	Reference #
COVID-19 patients	Spontaneous. Sputum was diluted in medium and a few droplets pipetted on glass slides, dried and fixed in 80% acetone.	Parallel reaction monitoring MS Proteomics	Determining the LOD of viral proteins in sputum and nasopharyngeal swabs of patients	The method is suitable for the successful detection of viral infection in clinical specimens and can be used in clinical and diagnostics labs	[21]
COVID-19 patients	Spontaneous. Sputum was treated with or without 10 units of rhDNase Samples were cytocentrifuged and then prepared for immunostaining.	Data-independent acquisition MS	Identification of neutrophil extracellular traps (NETS) in sputum and blood plasma of patients	Several NET-derived proteins were identified	[22]

**Table 2:** List of articles dealing with proteomics of sputum in COPD patients.

Subjects Investigated	Method of Sputum Collection and Processing	Proteomic Technique Applied	Target of the Rersearch	Finding	Reference #
Nonsmokers/Smokers, COPD Patients with or without emphysema	Induced *. Sputum was treated with DTT, and cellular and aggregated material removed. Proteins in the supernatant were alkylated and digested with trypsin.	Capillary LC-MS/MS	Finding biomarkers of emphysema	Mucins, long palate lung nasal clone 1 and other proteins were identified as potential biomarkers	[7,23]
Nonsmokers/Smokers, e-cigarette users	Induced. Sputum was diluted 1:1 in GuHC and proteins alkylated and digested with trypsin.	Nano LC /MS	Potential adverse health effects of e-cigarettes on human airways	The use of e-cigarette altered the profile of innate defense proteins in airway secretions	[24]
Never smokers, former smokers, current asymptomatic smokers, and smokers with early-stage COPD	Induced. Sputum was centrifuged and supernatant containing the proteins treated as indicated above (see ref. [7]).	Nano LC-MS/MS	Relationship between cigarette smoking and onset of COPD	Alterations in mucin/trefoil proteins and a prominent xenobiotic/oxidative stress response in smokers	[25]
Nonsmokers/Smokers with and without COPD	Induced. Sputum was centrifuged and treated as indicated above (see ref. [24]).	2-DE and MS	Longitudinal changes of lung function in smokers with COPD	Secreted BPIFB1 was significantly elevated in sputum of COPD patients compared with that of smokers and non-smokers	[26]
Non-smokers, smokers without and with moderate COPD	Induced. Sputum was centrifuged and treated as indicated above (see ref. [24]).	2D-DIGE coupled to MS	Identification of proteins involved in COPD pathogenesis	Altered proteins, COPD-, AATD-, and IPF-specific were identified	[27,28]
Patients with stable-COPD and acute exacerbation	Induced. Sputum was centrifuged and supernatant used for experiments.	Luminex-based multiplex equipment	Explore the percent of patients in which COPD is principally driven by activation of the interleukin-1β signalling pathway	A 2-fold increase of five IL-1β-mediated proteins was observed in sputum of acute exacerbation patients relative to stable COPD	[32]
Stable COPD patients and during exacerbations	Induced. Sputum was treated with DTT, and cellular and aggregated material removed. Proteins in the supernatant were alkylated and digested with trypsin.	Nano HPLC coupled to MS	Identify the substrates of proteases and determine their activity in airways of COPD patients	Differences in activity of airway elastase and processing of specific elastase substrates during stable disease and in the phase of exacerbation.	[33]
Patients with COPD and asthma	Induced. Sputum was centrifuged and petides contained in the supernatant identified by MS.	MALDI-TOF MS platform	Elucidate the mechanisms underlying the pathobiology of COPD and asthma	Peptide profiles allowed to differentiate COPD from asthma	[9,11,12,35]
Healthy controls, COPD, asthma, and COVID-19 patients	Spontaneous/induced. Sputum was diluted in PBS, centrifuged and proteins reduced with DTT followed by alkylation and digestion with trypsin.	UPLC-MS/MS	Finding of differentially expressed proteins specific to each group of patients	Proteomic changes specific for COVID-19 patients and different from those related to COPD and asthma	[36,37]

* In all cases of induced sputum (Table 2, Table 3, Table 4 and Table 5), induction was performed after inhalation of hypertonic (0.9% to 4.5%) saline with a nebulizer according to the procedure described in Section 3.

**Table 3:** List of articles dealing with proteomics of sputum in patients with asthma.

Subjects Investigated	Method of Sputum Collection and Processing	Proteomic Technique Applied	Target of the Rersearch	Finding	Reference #
Patients with controlled asthma and severe uncontrolled asthma	Induced. Sputum was centrifuged and loaded on 2-DE.	2 DE coupled to MALDI-TOF	Identifying biomarkers to differentiate the two conditions	S100 calcium binding protein A9 was considered a potential biomarker of neutrophilic inflammation in severe UA	[38]
Non-smokers; healthy non-smokers; ex-smokers and current smokers	Induced. Sputum plugs were separated into cells and supernatant. This latter was submitted to MS analysis.	LC-MS	Define severe asthma molecular phenotypes	The differentially expressed proteins identified allowed to distinguish current smokers from ex-smokers	[39]
Patients with moderate-to-severe asthma	Induced. Sputum plugs were selected and liquefied with DTT.Transcriptomic analysis was performed on extracted RNA from sputum cells derived from cell pellets	Affymetrix HT HG-U133 + PM GeneChip	Stratify patients into clusters	Four clusters were identified that showed significant differences in sputum proteomics and transcriptomics	[40]
Asthmatic patients	Induced. Sputum was treated with DTT, and cellular and aggregated material removed by centrifugation. Proteins in the supernatant were alkylated and digested with trypsin.	LC-MS	Patient stratification	Patients were stratified in 10 clusters representing 3 sub phenotypes of asthma: highly eosinophilic, highly neutrophilic, and highly atopic with relatively low granulocytic inflammation	[41]
Mild/moderate asthmatics, healthy controls and of a subset of severe asthmatics	Induced sputum was acquired and processed DTE as a mucolytic to obtain supernatant for mass spectrometric analysis.	LC-MS	Identify proteins associated with Gastro-oesophageal reflux disease (GORD) in asthmatic patients	GORD was three- and ten-fold more prevalent in severe asthmatics compared to mild/moderate asthmatics and healthy controls	[42]

**Table 4:** List of articles dealing with proteomics of sputum in patients with cystic fibrosis.

Subjects Investigated	Method of Sputum Collection and processing	Proteomic Technique Applied	Target of the Rersearch	Finding	Reference #
Exacerbated CF subjects, clinically stable CF children, and control subjects	Induced sputum was solubilized in the presence of a protease inhibitor cocktail to prevent proteolytic degradation and submtted to 2-DE.	2-DE coupled to MALDI-TOF MS	Comparison of sputum proteins among groups and capture of antigens from complex proteomes of sputum	Changes in protein profiles and expression were observed as markers of disease progression	[43,44]
Healthy subjects and patients with asthma, COPD, CF, and bronchiectasis	Induced. Sputum plugs were selected and processed with DTE after which PBS was added. Samples were filtered and centrifuged to remove the cells. Supernatants were submitted to MS analysis.	SELDI-TOF MS	Identification of potential biomarkers of suppurative and inflammatory lung disease	Potential biomarkers that differentiated each of the disease groups from healthy controls were identified	[45]
CF patients	Spontaneous.Sputum was homogenized in PBS with the addition of EDTA and of a protease inhibitor cocktail and split in aliquots for analysis.	2-DE coupled to MS/MS	Insight into the pathophysiology of the microbial community in lungs of CF patients	An important basis for future investigations on the physiology of microbial pathogens in CF in vivo	[46]
Healthy subjects and CF adults chronically infected with *P. aeruginosa*	Induced. The cell population was harvested from mucus plugs, washed and proteins extracted and treated as above indicated (see ref. [41]).	MudPIT platform	Identification of sputum cellular proteins	The CF proteome was largely distinct from that of healthy subjects	[47]
CF patients	Spontaneously expectorated sputum was homogenized by mixing with PBS containing DTE. The homogenate was digested with trypsin and submitted to analysis.	LC-MS/MS	Study of the *P. aeruginosa* physiology	Development of future treatment strategies aimed at altering PA physiology in vivo	[48]
CF patients	Spontaneous. Sputum plugs were washed and soluble proteins isolated.	1-DE and MS	Comparison of Several PA isolates from CF patients’ sputum	In a conventional medium, PAO1 expressed numerous proteins that were absent in the CF isolates	[49]

**Table 5:** List of articles dealing with proteomics of sputum in patients with lung cancer.

Subjects Investigated	Method of Sputum Collection and Processing	Proteomic Technique Applied	Target of the Rersearch	Finding	Reference #
Lung cancer patients and healthy controls	Induced. Sputum was centrifuged and supernatant diluted with PBS and filtered. Supernatant was submitted to 1-DE.	1-DE coupled to MS	Identification of proteins involved in lung cancer	The expression level of five proteins was higher in sputum of cancer patients compared to controls	[50]
Lung cancer patients and healthy controls	Spontaneous/induced. Diluted with normal saline and centrifuged to separate pellet from supernatant. This latter was collected and separate aliquots were saved for measurements.	ELISA assay	Establish a new diagnostic and prognostic biomarker for lung cancer	A significant increase in the level of MMP-2 was observed in the sputum of metastatic compared to the non-metastatic group	[51]
Smokers, subjects exposed to asbestos, and lung cancer patients	Induced sputum was centrifuged and potential cancer biomarkers identified in supernatant.	2D-DIGE coupled to LC-MS/MS	Detect protein abundance differences in the proteome of induced sputum obtained from different groups	Five proteins, four of which associated to lung cancer (PRDX2, TXN, GAPDH and S100A8), have been validated	[52]
Lung cancer patients	Spontaneous. DTT was added and pellet and supernatant were separated by centrifugation. Supernatant was filtered, concentrated and submitted to electrophoresis.	1-DE followed by nano LC-MS/MS	Identification of patients who are most likely to respond to platinum-based lung cancer chemotherapies	UDP-glucose glycoprotein glucosyltransferase 1 and collagen chain alpha-1 (VI) were best biomarker candidates for sensitivity to cisplatin	[53]

## Abbreviations

Chronic obstructive pulmonary disease	COPD
Cystic fibrosis	CF
Parallel reaction monitoring	PRM
Reactive oxygen species	ROS
Neutrophil extracellular traps	NETs
Idiopathic pulmonary fibrosis	IPF
Alpha1-antitrypssin deficiency	AATD
Controlled asthma	CA
Severe uncontrolled asthma	SUA
Ex-smokers	ESA
Current smokers	CA
Gastro-oesophageal reflux	GORD
Forced expiratory volume in 1 sec	FEV_1_
